# Books Reviewed

**Published:** 1867-03

**Authors:** 


### Books Reviewed.

The Renewal of Life—Lectures, chiefly Clinical, by THOMAS KING CHAMBERS, M. D., Honorary Physician to H. R. H. the Prince of Wales, Consulting Physician and Lecturer on the Practice of Medicine at St. Mary's Hospital, Consulting Physician to the St. Luke's Hospital. Second American from the fourth London edition. Philadelphia: Lindsay & Blakiston, 1866.

The early appearance of the second American edition of this work clearly demonstrates the estimation in which it is held by the profession ; and it is with pleasure that we repeat our expressions of approbation of a work, so full of practical suggestions, clinical observations and philosophical reasonings. No new additions to this issue have been made, owing to an illness of the author, but the same has been thoroughly revised and re-arranged. In design and scope this work is unsurpassed, treating the subjects under consideration in a masterly and scientific style, and according to the present elevated standard of medical opinion, pointing out the errors of the past. The author rightfully considers physiology as the foundation of all medicine, and in his "*L'nvoi*" presses upon the students the importance of considering this branch as the "key-stone and binding link of all their knowledge, and the firm foundation which they are to crown with their future practice." "It is an error," he says, "deadly to the usefulness of our professiou, to say or to do anything toward fostering an idea that the organic laws of health and disease are different; it is still worse to paint them in opposition. On the modern principles of dividing labor we have separate lectures on anatomy, physiology, pathology, and the practice of medicine; but both instructors and pupils should never lose sight of these as branches of one study, as being in truth all limbs of the same tree; and those who follow them, one after the other should still pursue the same end—a knowledge of man's nature with a view to the culture of his physical well-being."

To the student just entering upon his profession, and to the practitioner of years' standing, this work of Dr. Chambers will prove highly instructive and afford a satisfactory explanation of many perplexing questions daily presenting themselves in the pursuit of their labors.

---

On the Functions and Disorders of the Reproductive Organs, in Childhood, Youth, Adult Age and Advanced Life, considered in their Physiological, Social and Moral Relations. By WILLIAM ACTON, M. R. C. S., late Surgeon to the Islington Dispensary, and formerly Externe to the Venereal Hospitals, Paris; Fellow of the Royal Med. and Chir. Society, etc., etc. Second American, from the fourth London edition. Philadelphia: Lindsay & Blakiston, 1867.

Mr. Acton, in discussing the functions and disorders of the reproductive organs has treated this subject in the calmest and most philosophic spirit, his language being as choice and delieate as the nature of his thema would allow without becoming ambiguous. The author has for many years directed his especial attention to the study of tho generative organs, and has furnished this work as the result of a life's close observation. All circumstances appertainiug to the organs in question have been closely investigated and carefully recorded. Too long have men of science allowed themselves to ignore this subject, owing either to a perverted sense of delicacy or to a repugnance of approaching a subject of this nature, thus exposing in a great measure the unfortunate and misguided victims of passion to the nefarious tricks of advertising scoundrels. Mr. Acton has conferred a great favor upon the profession in so manfully attacking long-established abuses, and we would welcome him as a pioneer in a field of medical literature, thus far almost totally neglected.

We would especially invite the attention to the chapters considering the management of childhood and youth, to guard against the acquisition of that loathsome habit, masturbation. Not alone will physicians derive many valuable hints, aiding them in the treatment of disease at that period of life, but also parents and instructors will become better adapted to the management of their offspring and to the charges intrusted to their care.

